# Complexation and Thermal Stabilization of Protein–Polyelectrolyte Systems via Experiments and Molecular Simulations: The Poly(acrylic acid)/Lysozyme Case

**DOI:** 10.3390/polym17152125

**Published:** 2025-08-01

**Authors:** Sokratis N. Tegopoulos, Sisem Ektirici, Vagelis Harmandaris, Apostolos Kyritsis, Anastassia N. Rissanou, Aristeidis Papagiannopoulos

**Affiliations:** 1 School of Applied Mathematical and Physical Sciences, National Technical University of Athens, GR-15772 Athens, Greece; stegopoulos@mail.ntua.gr (S.N.T.); akyrits@central.ntua.gr (A.K.); 2 Computation-Based Science and Technology Research Center, The Cyprus Institute, Nicosia 2121, Cyprus; s.ektirici@cyi.ac.cy (S.E.); harman@uoc.gr (V.H.); 3 Department of Mathematics and Applied Mathematics, University of Crete, GR-71409 Heraklion, Greece; 4 Institute of Applied and Computational Mathematics, Foundation for Research and Technology Hellas, IACM/FORTH, GR-71110 Heraklion, Greece; 5 Theoretical & Physical Chemistry Institute, National Hellenic Research Foundation, 48 Vassileos Constantinou Avenue, GR-11635 Athens, Greece

**Keywords:** protein–polyelectrolyte complexation, light scattering, thermal stabilization, molecular dynamics simulations, atomic information

## Abstract

Protein–polyelectrolyte nanostructures assembled via electrostatic interactions offer versatile applications in biomedicine, tissue engineering, and food science. However, several open questions remain regarding their intermolecular interactions and the influence of external conditions—such as temperature and pH—on their assembly, stability, and responsiveness. This study explores the formation and stability of networks between poly(acrylic acid) (PAA) and lysozyme (LYZ) at the nanoscale upon thermal treatment, using a combination of experimental and simulation measures. Experimental techniques of static and dynamic light scattering (SLS and DLS), Fourier transform infrared spectroscopy (FTIR), and circular dichroism (CD) are combined with all-atom molecular dynamics simulations. Model systems consisting of multiple PAA and LYZ molecules explore collective assembly and complexation in aqueous solution. Experimental results indicate that electrostatic complexation occurs between PAA and LYZ at pH values below LYZ’s isoelectric point. This leads to the formation of nanoparticles (NPs) with radii ranging from 100 to 200 nm, most pronounced at a PAA/LYZ mass ratio of 0.1. These complexes disassemble at pH 12, where both LYZ and PAA are negatively charged. However, when complexes are thermally treated (TT), they remain stable, which is consistent with earlier findings. Atomistic simulations demonstrate that thermal treatment induces partially reversible structural changes, revealing key microscopic features involved in the stabilization of the formed network. Although electrostatic interactions dominate under all pH and temperature conditions, thermally induced conformational changes reorganize the binding pattern, resulting in an increased number of contacts between LYZ and PAA upon thermal treatment. The altered hydration associated with conformational rearrangements emerges as a key contributor to the stability of the thermally treated complexes, particularly under conditions of strong electrostatic repulsion at pH 12. Moreover, enhanced polymer chain associations within the network are observed, which play a crucial role in complex stabilization. These insights contribute to the rational design of protein–polyelectrolyte materials, revealing the origins of association under thermally induced structural rearrangements.

## 1. Introduction

Protein–polyelectrolyte complexes are increasingly investigated for their role in developing advanced materials across biomedical sectors, e.g., in the selective isolation of active antibody fragments [1]; biotechnological sectors, e.g., through the enhancement of protein stability and delivery through protection, controlled release, and functionality improvement [2]; and food science sectors, e.g., through the microencapsulation of oils and flavors in foods and beverages [1], stabilizing air/water interfaces by forming strong viscoelastic films that reduce gas permeability, enhancing foam stability, and improving the texture and creaminess of food products [3]. Self-assembled nanosized complexes show promise in drug delivery, e.g., through protein encapsulation via polypeptide complex coacervation [4] and the protection of drugs from premature degradation [5]; in tissue engineering, e.g., through the restoration of impaired bone and skeletal tissue [6]; and in therapeutics, e.g., through wound covering for patients suffering extensive burns [7], wound healing [8], and food applications [5,9,10,11,12]. This is mainly due to their ability to encapsulate bioactive molecules [13] and release them in a controlled or stimuli-responsive way [14]. These systems can be engineered to respond to environmental stimuli such as pH, temperature, or ionic strength, enabling the development of “smart” drug delivery platforms [15,16]. Among these, protein–polyelectrolyte complexes serve as versatile building blocks for constructing functional nanomaterials with tailorable properties [17,18]. A key advantage of self-assembled protein–polyelectrolyte nanoparticles (NPs) is their preparation, which involves no toxic substances, their biocompatibility, and biodegradability [5,11,19,20,21]. Proteins bring multifunctionality as they have hydropathy surface distribution and pH-tunable surface charge distribution in addition to their specific functionality [22].

Specifically, in protein–ionic polysaccharide complex (NPs), it has been shown that stabilization against pH changes, to regimes where the protein and polysaccharide electrostatically repulse, can be achieved in a biocompatible manner. Mild thermal treatment promotes irreversible hydrophobic associations between protein molecules within the NPs. These associations are not influenced by pH changes [18,21,22,23]. A very well-studied example is bovine serum albumin (BSA). At elevated temperature, BSA exposes hydrophobic amino acids, allowing β-sheet formation among different BSA molecules upon cooling [24]. This effect has been applied to chondroitin sulfate/BSA electrostatic complexes to hinder dissociation at neutral pH [18].

Poly(acrylic acid) (PAA) can form electrostatically stabilized complexes with positively charged proteins, which leads to nanoscale structures. Its stability across a range of conditions and responsiveness to pH make it ideal for biomedical formulations [25,26]. One particularly attractive protein for NP development is lysozyme (LYZ), a naturally occurring globular enzyme with a well-established role in antimicrobial defense. LYZ acts by hydrolyzing the peptidoglycan layer of bacterial cell walls and has demonstrated strong antibacterial activity, especially against Gram-positive bacteria [27,28,29,30]. Additionally, it possesses antiviral, antifungal, anti-inflammatory, anticancer, and immunomodulatory properties, making it a valuable candidate for pharmaceutical and therapeutic applications [28,31]. LYZ has traditionally been a model protein for protein–polyelectrolyte complexes [32]. Moreover, LYZ is relatively stable under thermal treatment. For example, it keeps its structure at 328 K for 60 min [33]; at 338 K, 55% of its native conformation unfolds for the same period, whereas at 348 K, the effect is significantly higher. Mild denaturation of LYZ above 343 K induces self-aggregation and phase separation [34]. As with BSA, LYZ aggregation upon thermal treatment involves disruption of intra-protein and formation of inter-protein disulfide bonds [35].

The temperature-dependent behavior of LYZ has been extensively investigated through molecular dynamics (MD) simulations [36,37,38,39] and experiments [40,41,42,43,44], which have shown that both aggregation and conformational changes intensify at elevated temperatures. Using all-atom and coarse-grained MD simulations, Islam et al. showed that LYZ undergoes more pronounced conformational changes and forms more compact aggregates at 340 K, while at 300 K, the structural changes are primarily due to aggregation rather than thermal effects [39]. Kaumbekova and Shah, using MD simulations, reported that LYZ maintains a compact structure and demonstrates partial refolding capability when thermally unfolded and subsequently cooled in Deep Eutectic Solvent (DES) environments, suggesting that controlled thermal modulation can enhance structural stability [45]. Similarly, a simulation study by Huda et al. revealed that LYZ retains its compactness across a broad temperature range (270–380 K), with only slight increases in flexibility at higher temperatures [46]. While simulated thermal processes have been widely employed to investigate protein folding and unfolding mechanisms [47,48,49,50,51], their impact on the protein’s interactions with other macromolecules, such as polymers, remains largely unexplored, particularly in multi-molecular systems.

In our earlier study, we investigated how the secondary structure of a single LYZ molecule is affected by its interaction with multiple PAA chains under different temperature conditions at physiological pH (pH 7), focusing on the fundamental molecular origins of protein–polymer association [52]. Building on these insights, we subsequently extended the analysis to multi-molecular systems comprising multiple LYZ and PAA molecules, systematically exploring how variations in pH and temperature influence the complexation process and drive network formation between proteins and polyelectrolytes [53]. In the present study, we advance this framework by applying a temperature-controlled quenching protocol to multi-molecular LYZ-PAA systems at different pH values. Our aim is to elucidate the molecular mechanisms underlying the irreversible interactions induced by thermal treatment, which confer stability to protein–polyelectrolyte nanostructures even under conditions where both LYZ and PAA are negatively charged (pH 12).

Using an integrated experimental-simulation approach, we investigate LYZ-PAA network formation and elucidate the atomic details of the irreversible structural changes induced by thermal treatment. SLS and DLS are used to confirm the formation of NPs at neutral pH, the structural changes upon pH increase, and the effect of thermal treatment on stability upon pH changes. FTIR was used to evaluate molecular interactions and CD to monitor secondary structure changes in LYZ within the nanostructures with PAA. Atomistic molecular dynamics simulations allow us to explore the atomistic information behind the structural reorganization within the protein–polymer complexes and its effect on the interactions, imposed by a gradual thermal reduction. The complementarity of experimental and simulation findings provides crucial knowledge for developing and refining biomaterials with specific, controllable characteristics.

## 2. Systems and Methods

### 2.1. Experimental Details

#### 2.1.1. Materials and Sample Preparation

LYZ from chicken egg white (powder form) and PAA with a molecular weight of approximately 4000 g mol^−1^ (PDI ~2) 45 wt.% aqueous solution were purchased from Sigma-Aldrich (Burlington, MA, USA). Stock aqueous solutions of each component were prepared at a concentration of 1 mg mL^−1^ using distilled water. Final concentrations and PAA/LYZ mass ratios (r_m_) were obtained by mixing appropriate volumes of stock solutions with distilled water under gentle stirring. Complex formation was carried out at room temperature (298 K). The mixing procedure was as follows: PAA was first added to distilled water, followed by the addition of LYZ. In addition to the complexes, a control sample containing only the protein (r_m_ = 0) was also prepared to allow for the investigation of the effect of PAA on the secondary structure of the protein and to assess the strength of the interaction between the polyelectrolyte and the protein. The total volume of each sample was 1 mL in all cases. The pH was adjusted to the preferred values 7 or 12 through the addition of NaOH (1 M). It was measured using pH indicator papers with an accuracy ± 0.2 pH units as confirmed by measurements with a pH meter in solutions of 10 mL sample volumes.

Thermal treatment was performed at pH 7 by placing Eppendorf tubes containing the samples in an oven at 353 K for 30 min and then leaving them at 298 K until equilibration. This specific temperature for thermal treatment was chosen as it lies slightly above the denaturation temperature of LYZ (348 K) [33], thereby promoting partial unfolding and potential structural rearrangements of the protein. All experiments were performed at 298 K. Each experiment was conducted a minimum of three times, and the results are reported as mean values with corresponding standard deviations.

#### 2.1.2. Static and Dynamic Light Scattering

Light scattering (LS) experiments were conducted using an ALV/CGS-3 compact goniometer system (ALVGmbH, Langen, Hessen, Germany), equipped with an ALV-5000/EPP multi-tau digital correlator and a He-Ne laser operating at a wavelength of 632.8 nm. In Static Light Scattering (SLS), the Rayleigh ratio, R(q), was measured over a range of scattering angles from 30° to 120°. The scattering wave vector q (Equation (1)) is expressed as follows:

(1) $$q=\frac{4\pi n_{0}}{\lambda }sin\left(\frac{\theta }{2}\right)$$

where n_0_ is the solvent’s refractive index, λ is the laser wavelength, and θ is the scattering angle. The SLS data were analyzed using Equation (2):

(2) R(q)=KcMP(q) 

where M is the weight-averaged molar mass, c is the mass solution concentration, and K is the light scattering contrast factor K = $\;\frac{4\pi^{2}n_{0}^{2}}{N_{A}\lambda^{4}}{\left(\frac{\partial n}{\partial c}\right)}^{2}$. Here, N_A_ is the Avogadro number, and $\frac{\partial n}{\partial c}$ is the refractive index increment in the solute and solvent system. For mixtures of PAA and LYZ, ∂n/∂c was calculated as a weighted average:

$\left(\frac{\partial n}{\partial c}\right)=\frac{c_{PAA}}{C_{PAA}+C_{lysozyme}}{\left(\frac{\partial n}{\partial c}\right)}_{PAA}+\frac{c_{lysozyme}}{C_{PAA}+C_{lysozyme}}{\left(\frac{\partial n}{\partial c}\right)}_{lysozyme}$, with ${\left(\frac{\partial n}{\partial c}\right)}_{PAA}=0.145$ mL g^−1^ and ${\left(\frac{\partial n}{\partial c}\right)}_{lysozyme}=0.180$ mL g^−1^. Given that the calculated $\left(\;\frac{\partial n}{\partial c}\right)$ value was not lower than 0.177 even at the highest mass ratio (r_m_ = 0.1), a value of $\left(\;\frac{\partial n}{\partial c}\right)$ ≈ 0.18 mL·g^−1^ was assumed for all mixing ratios.

The Rayleigh ratio R(q) [54,55] describes the scattered light intensity and was interpreted using the modified Guinier approximation P(q) (Equation (3)). The quadratic term is used to account for the nonlinearity of the plots that may result from polydispersity and/or internal correlation effects [18]. Intraparticle light interference is important for NPs with R_g_ > 100–200 nm. However, Equation (3) captures the slope of the Guinier plot at very low q.

(3) $$P\left(q\right)=e^{-\frac{1}{3}q^{2}R_{g}^{2}+B{\left(q^{2}\right)}^{2}}$$

This relationship allows for the estimation of the radius of gyration, R_g_, of the scattering particles [54]. In dynamic light scattering (DLS), the autocorrelation functions of the scattered light intensity, g_2_(τ), were measured as a function of lag time τ [55]. Using the Siegert relation $g_{2}(\tau )-1={\beta |g_{1}\left(\tau \right)|}^{2}$, where β is a normalization constant, the field autocorrelation function g_1_(τ) is calculated. To obtain particle size distributions, the field correlation function g_1_(τ) was extracted using CONTIN analysis to derive the distribution of characteristic relaxation times τ_c_. These were converted to diffusion coefficients D using D=1τcq2. The hydrodynamic radius, R_h_, was then calculated using the Stokes–Einstein relation that connects the diffusion coefficient D with the viscosity of the solvent viscosity η, and the temperature (Equation (4)).

(4) $$R_{h}=\frac{k_{\beta }T}{6\pi hD}$$

#### 2.1.3. Fourier Transform Infrared Spectroscopy

Fourier transform infrared (FTIR) spectroscopy measurements were performed using a Bruker Equinox 55 spectrometer (Bruker, Ettlingen, Germany) equipped with a diamond-attenuated total reflectance (ATR) accessory (SENS-IR Technologies, Danbury, CT, USA). Approximately 10 μL of each sample solution was deposited at the center of the ATR crystal and subsequently dried under a gentle stream of nitrogen gas to form a thin film. Spectra were collected over the wavenumber range of 500–5000 cm^−1^ at a resolution of 2 cm^−1^, and 64 scans were averaged for each measurement.

#### 2.1.4. Circular Dichroism

Circular dichroism (CD) spectra were acquired using a Jasco J-815 spectropolarimeter (JASCO Corporation, Tokyo, Japan) equipped with a PTC-423S/15 Peltier temperature control unit. Samples were placed in 1 mm path length quartz Suprasil cuvettes. The aqueous solutions were diluted to achieve a final LYZ concentration of 0.1 mg mL^−1^, identified as optimal for generating a sufficient CD signal. For each sample, spectra were obtained by averaging four consecutive scans. CD spectra were recorded in the region of 185–300 nm, and the data retrieved in units of mdeg were converted into molar ellipticity units Δ*ε* of M^−1^ cm^−1^ (Equation (5)).

(5) $$\Delta \varepsilon =\theta \cdot \frac{0.1\cdot MRW}{\left(P\cdot c\right)\cdot 3298}$$

where θ is the measured ellipticity in mdeg, MRW is the mean residue weight, Δ*ε* is the molar ellipticity per residue in M^−1^ cm^−1^, c is the LYZ concentration in mg mL^−1^, and P is the path length in cm. The protein secondary structure was interpreted and quantified using the open source Beta Structure Selection (BeStSel, v1.3.230210) analysis tool [56].

#### 2.1.5. Electrophoretic Light Scattering

Electrophoretic light scattering (ELS) measurements were performed using a Zetasizer Nano-ZS (Malvern Instruments Ltd., Malvern, UK). The instrument determines the electrophoretic mobility of particles (Equation (6)) at a fixed backscattering angle of θ = 173°, which is subsequently converted into zeta potential (*ζ*_p_) values (Equation (7)). For each sample, ten measurements were taken at room temperature, and the mean *ζ*_p_ along with the standard deviation was calculated. In all cases, single peaks with moderate polydispersity were observed for the *ζ*_p_ distributions. The *ζ*_p_ values were obtained using Henry’s equation and the Smoluchowski approximation, under which the Henry function f(R/λ_D_) is assumed to be constant and equal to 3/2 [57], where *R* is the nanoparticle radius and *λ_D_* is the Debye length.

(6) μe=veE=23εrε0ζf(RλD)η

(7) $$\zeta_{p}=\frac{\eta \mu_{e}}{\varepsilon_{r}\varepsilon_{0}}$$

In Equations (6) and (7), v_e_ is the drift velocity of the NP, E is the electric field, *ε*_r_ and *ε*_o_ are the relative dielectric permittivity of the solvent and the dielectric permittivity of the vacuum, respectively, and η is the viscosity of the solvent [58,59].

#### 2.1.6. UV–Visible Spectroscopy

UV–Vis absorption spectra were recorded using a Perkin Elmer Lambda 19 UV–Vis–NIR spectrophotometer. Samples were loaded into quartz cuvettes with a 1 cm optical path length. Spectra were collected over the wavelength range of 220–800 nm to characterize the absorbance profile of LYZ. The absorption maximum, λ_max,_ was observed at approximately 280 nm, which is consistent with the typical UV-Vis absorption spectrum of LYZ due to the presence of aromatic amino acids like tryptophan and tyrosine [60].

### 2.2. Simulation Details

Table 1 contains details for the total number of atoms in the systems, water atoms, protein atoms, and ions. All simulations were performed in a cubic box with dimensions of (16 × 16 × 16) nm^3^.

Protein–polymer mixtures were simulated in an aqueous environment, using all-atom MD simulations and an explicit solvent model, under two different pH values, 7 and 12, and at temperatures of 298 K, 368 K, and 298 K after quenching from 368 K. pH 7 represents physiological conditions, while pH 12 was chosen to investigate how the deprotonation of LYZ affects its electrostatic interactions with the polymer and alters the stability of the complex. The mass ratio, m_PAA_/m_LYZ_, in the mixture was adjusted to 0.1 by mixing 16 LYZ molecules and 8 PAA chains, each consisting of 40 monomer units. This is the optimal mass ratio for complex NPs suggested by the experiments. The PAA chains were modeled assuming 50% deprotonation of carboxylic groups, based on an effective pKa shift in polyelectrolyte environments (pKa~4.5) [61], resulting in 20 negative charges per 40-monomer chain. This is consistent with Manning’s theory, which predicts counterion condensation due to the charge spacing (0.27 nm) and Bjerrum length (0.7 nm at 298 K) [62], effectively reducing the linear charge density. Simulations of 300 ns were conducted for all systems. To examine the effect of thermal treatment on protein–polymer interactions, a quenching process was applied to selected systems, where the temperature was gradually decreased from 368 K to 298 K at a rate of approximately 0.78 K/ns over a period of 90 ns. We employed the high-resolution crystal structure 1GWD of hen egg white lysozyme (1.7 Å), which includes the entire amino acid sequence, as the basis for our MD simulations, after removing all non-protein ligands. The protonation states of LYZ residues were explicitly adjusted for each pH using pKa-based calculations in CHARMM-GUI [63], setting a fixed charge distribution that reflects the dominant ionization state under the given conditions. This approach incorporates the pH dependence of electrostatic interactions via static protonation, which, although it does not dynamically respond to the local environment, is widely adopted for modeling pH effects in protein–polyelectrolyte systems.

The residues that were protonated are detailed in our previous work [53]. Counter ions (Na^+^ and Cl^−^) were added in order to neutralize the system and to set the ionic strength of the solution to I = 0.15 M (Table 1). All-atom MD simulations were carried out using the GROMACS 2023 simulation package [64], employing the all-atom AMBER99SB-ILDN force field [65,66], which is a widely used and well-regarded force field for protein simulations and is known to accurately capture protein dynamics [67,68,69,70,71]. The SPC/E water model [72,73] was used to explicitly represent water molecules. Simulations were performed in the NPT ensemble using the leap-frog integrator with a 1 fs time step. Pressure coupling was implemented using the Berendsen barostat [74] with isotropic scaling, a time constant of 1.0 ps, and a reference pressure of 1.0 bar. Temperature was regulated using the velocity-rescale thermostat [75], with separate coupling groups defined for the protein and non-protein components. Short-range electrostatic and van der Waals interactions were truncated at 1.0 nm, while long-range electrostatic interactions were handled using the Particle Mesh Ewald (PME) method with a Fourier grid spacing of 0.16 nm and fourth-order cubic interpolation. Dispersion corrections were applied to both energy and pressure to account for long-range truncation effects. Periodic boundary conditions were enforced in all spatial directions.

## 3. Results and Discussion

### 3.1. Experimental Results

Several screening experiments were conducted in a range of mass ratios (r_m_ = c_PAA_/c_LYZ_), specifically 0.001, 0.003, 0.01, 0.03, 0.1, 0.3, 0.5, 0.7, and 1. This initial assessment was necessary to determine the optimal conditions for the formation of well-defined PAA/LYZ NPs. The mass ratios r_m_ = 0.003, 0.01, 0.03, and 0.1 were identified as the most suitable for further investigation, as they provided consistent NP formation with size in the range of 100–200 nm and large molar masses (>2 × 10^7^ g mol^−1^). The complexation was tested at pH 7, whereas the stability at repulsive conditions was tested at pH 12. The isoelectric point of LYZ is pI_LYZ_ = 10.7, and therefore, these two pH values represent conditions of attractive and repulsive PAA/LYZ electrostatic interactions.

It is well established that strong complexation between a protein and a polyelectrolyte occurs under stoichiometric charge neutralization conditions [13,18,21,22]. LYZ has a molecular weight of 14.300 g mol^−1^ [76] and carries a net positive charge of +8 at pH 7 [77]. Poly(acrylic acid) (PAA) has a molecular weight of 4000 g mol^−1^ and a weight-average number of 56 monomers. The average chain length of PAA in the experimental systems (56 monomers) is slightly greater than in the simulated systems (40 monomers), but this difference is not expected to impact the system’s behavior. Assuming a charge of −0.5 per PAA monomer at pH 7, charge neutrality is expected at a mass ratio (r_m_) of approximately 0.08. This value lies within the range of mass ratios selected for further investigation (r_m_ = 0.003, 0.01, 0.03, 0.1), and it served as a reference point for identifying the conditions under which optimal nanocomplex formation is expected.

Separate protein–polyelectrolyte solutions were prepared for every mass ratio (r_m_). This approach enabled the application of distinct processing conditions (e.g., pH adjustment, thermal treatment) and facilitated the use of multiple complementary experimental techniques, while ensuring precise knowledge and reproducibility of the exact mass ratio in each case.

**Figure 1:**
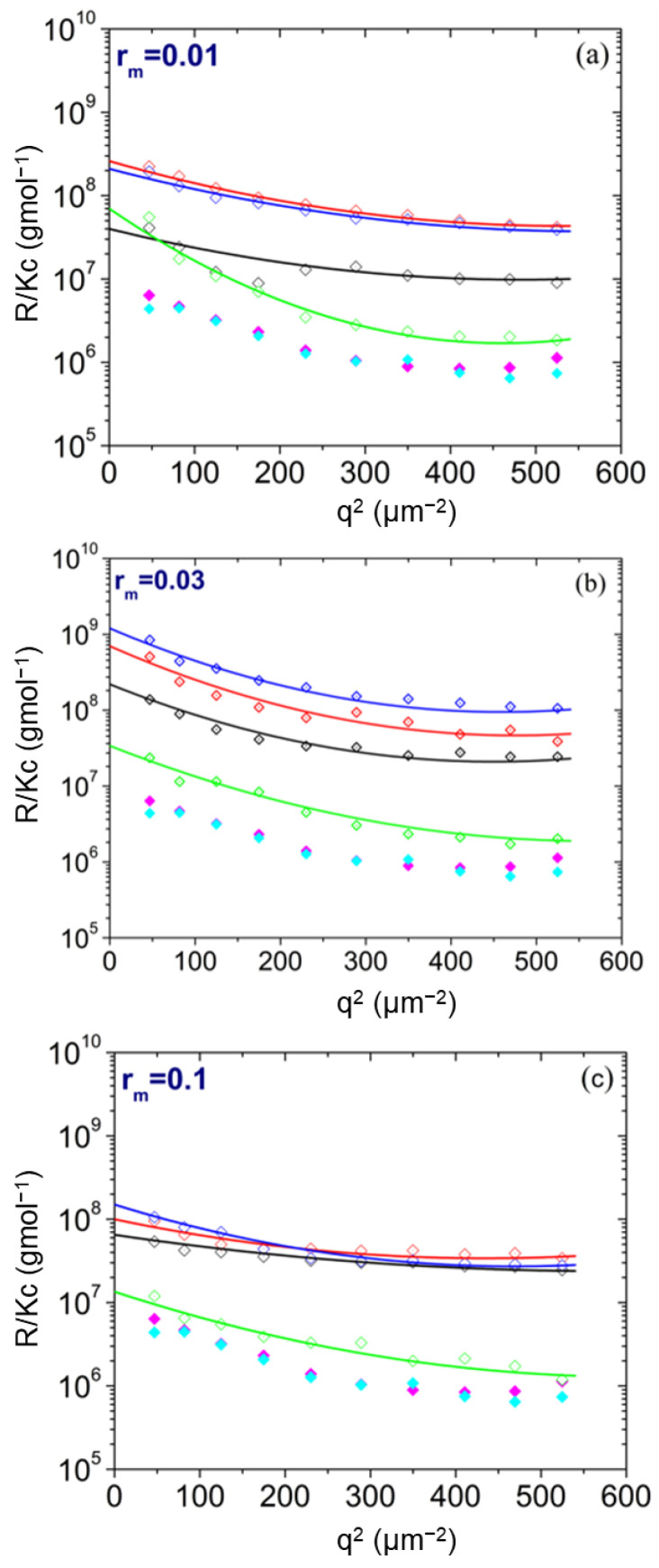
Guinier plots of PAA/LYZ complexes at (**a**) r_m_ = 0.01, (**b**) r_m_ = 0.03, and (**c**) r_m_ = 0.1. Conditions: pH 7 (black), thermally treated (TT) at pH 7 (red), pH 12 (green), and at pH 12 after thermal treatment at pH 7 (blue). Reference plots for pure LYZ (magenta) and pure PAA (cyan) (at pH 7) are also included. Fitted data from the complexes with Equation (3) are included as lines.

Guinier plots obtained from PAA/LYZ complexes for different mass ratios (r_m_ = 0.01, 0.03, and 0.1) at pH 7 and pH 12 with or without thermal treatment are shown in Figure 1. For reference, the scattering data of pure LYZ and pure PAA (at pH 7) are also included. Notably, for all r_m_ values and conditions, the complexes’ scattering intensity is significantly higher than that of the individual components, indicating the formation of complex NPs with high molar mass. This enhancement in scattering suggests strong electrostatic associations between the positively charged LYZ and the negatively charged PAA chains, even at relatively low PAA concentrations. The increased signal observed across all Guinier plots confirms the formation of nanostructured complexes, regardless of the mixing ratio. Nevertheless, it should be noted that maximum intensity is observed for the samples with mass ratio r_m_ = 0.03.

The thermally treated (TT) samples exhibited significantly stronger scattering intensity than the untreated ones. This observation suggests that heating promotes further association of free (uncomplexed) protein on the NPs due to the expected partial unfolding that drives LYZ-LYZ association. At pH 12, the scattered intensity of the NPs is significantly lower than that one at pH 7, showing that under conditions where PAA and LYZ have charges of the same sign, the NPs are substantially destabilized and disintegrated. In contrast, the TT NPs appeared virtually intact upon an increase in pH to 12, showing that thermal treatment stabilizes them against dissolution under conditions of PAA/LYZ electrostatic repulsion. The microscopic origin of this behavior and the reasons for stabilized versus destabilized structures are crucial questions that are extensively explored through atomistic simulations.

**Figure 2:**
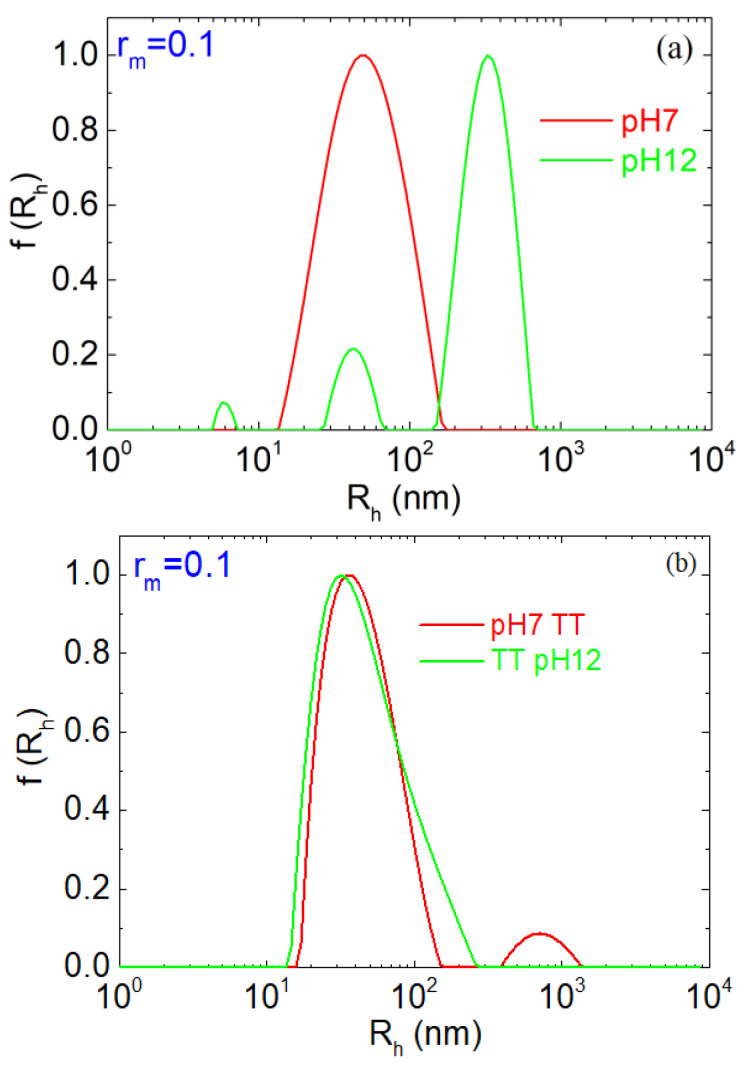
Hydrodynamic radius distributions via CONTIN analysis at 90° for PAA/LYZ complexes with r_m_ = 0.1 (**a**) at pH 7 (red) and pH 12 (green) and (**b**) after thermal treatment at pH 7 (red) and pH 12 (green).

Figure 2 presents the hydrodynamic radius distributions of the complexes at a mass ratio r_m_ = 0.1. At pH 7, NP solutions exhibit a dominant and well-defined peak at ~50–60 nm of a single population of NPs in solution. A secondary low-intensity peak appears only in the TT sample, suggesting minor aggregation. This overall similarity between treated and untreated samples indicates that thermal treatment does not perturb the particle size distribution.

In contrast, at pH 12, the behavior changes significantly. Without thermal treatment, three distinct populations are detected, with hydrodynamic radii centered at approximately 5 nm, 40–50 nm, and 330 nm. The first peak (5 nm) likely corresponds to LYZ aggregates, as the native hydrodynamic radius of LYZ is ~2 nm [78,79]. The second and third peaks represent PAA/LYZ NPs and larger complex aggregates that may be partially disintegrated complexes with open morphology, respectively. These are clear signs of NP disintegration. For TT NPs, the increase in pH from 7 to 12 does not result in such changes. The R_h_ distribution is close to the one at pH 7, confirming further the stabilization of NPs by thermal treatment. This behavior aligns with our previous works on the thermal stabilization of protein polysaccharide NPs [13,18,23,80]. Partially unfolded protein molecules within the NPs associate through the binding of exposed hydrophobic regions that self-assemble upon cooling. The overall thermostability of LYZ under physiological conditions and the local conformational changes induced by increased temperature have been extensively analyzed in our previous publication [52], where it was shown that certain types of secondary structures undergo a partially irreversible shift upon thermal treatment and different amino acids are positioned nearest to PAA, resulting in different PAA-LYZ interactions. However, experimental observations do not reveal direct details on the formation of contacts upon thermal treatment, e.g., PAA-LYZ associations that could support LYZ-LYZ hydrophobic contacts on the stabilization of the NPs. This is precisely where atomistic simulations, discussed in the following section, offer valuable insights. In conclusion, thermal treatment enhances nanoparticle stability at higher pH conditions that would typically lead to complex dissociation. This is in contrast to untreated samples, where strong electrostatic repulsion between the negatively charged components inhibits stable complexation. Further information on the hydrodynamic radius distribution is presented in Figure S1.

**Figure 3:**
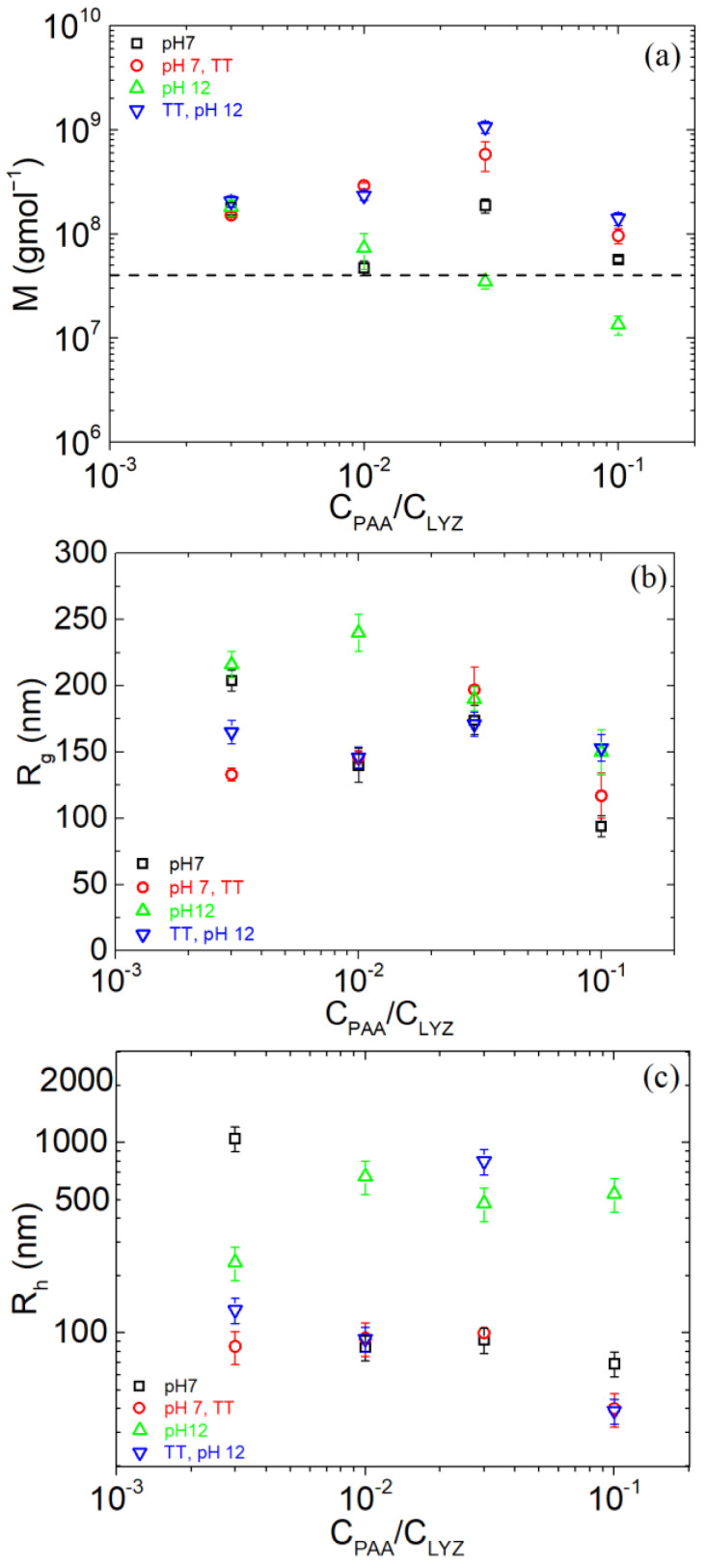
(**a**) Apparent molecular mass M_w_, (**b**) radius of gyration R_g_, and (**c**) hydrodynamic radius R_h_ (CONTIN analysis at 90°) at pH 7 (black), TT at pH 7 (red), pH 12 (green), and TT (at pH 7) at pH 12 (blue), of PAA/LYZ complexes.

In Figure 3, the extracted parameters from SLS and DLS as a function of r_m_ are presented. At pH 7, M values remain within the same order of magnitude across all compositions, indicating no clear dependence on the amount of added PAA. Furthermore, both untreated and TT samples at this pH exhibit similar M values, suggesting that thermal treatment has minimal impact on the complex formation under near-neutral conditions, as seen in the Guinier plots (Figure 1). In contrast, a distinct trend is observed at pH 12. The M values of the untreated samples decrease sharply with increasing PAA content. This behavior is likely attributed to strong electrostatic repulsion between LYZ and PAA, both of which carry negative charges at high pH. Particularly at r_m_ = 0.1, no complexation appears to occur, as the NPs disintegrate upon shifting the pH to 12. Remarkably, when samples undergo thermal treatment, the measured M values become nearly identical across all conditions and are pH-independent, as shown in the Guinier plots (Figure 1). The dashed line in Figure 3a indicates roughly the scattering from free LYZ and PAA to show that the scattering from NPs is significant.

Regarding the radius of gyration (R_g_), it is between 100 and 200 nm for all cases with no clear trend (Figure 3b). At pH 12, the untreated samples exhibit the highest R_g_ values across the range, indicating the formation of more extended or loosely connected structures, as expected from the DLS size distributions (Figure 2), which was attributed to electrostatic repulsion between the negatively charged LYZ and PAA chains. In contrast, TT samples at pH 12 display R_g_ values that closely align with those observed at neutral pH, suggesting that thermal treatment promotes the stabilization of the complexes as discussed above.

With respect to the hydrodynamic radius (Figure 3c), the effect of thermal treatment stabilization is clearly seen as R_h_ is mostly at about 100 nm or lower for all conditions except for untreated NPs at pH 12, where it is much higher, as discussed for r_m_ = 0.1 (Figure 2). The exceptionally high R_h_ found in r_m_ = 0.003 at pH 7 without thermal treatment may be attributed to very large and loosely connected complexes, as the PAA amount is very low. In the case of r_m_ = 0.03 at pH 12 after thermal treatment at pH 7, the large R_h_ (~800 nm) may signify the presence of clustered complexes that dominate the observed diffusion by DLS. However, R_g_ in this case is within the 100–200 nm scale, showing that the NPs exist in these solutions. Nevertheless, the case of r_m_ = 0.1 was selected as the most optimum one as the complexation, disintegration, and destabilization process was shown by all observed LS parameters, i.e., M, R_g_, and R_h_, and size distributions.

**Figure 4:**
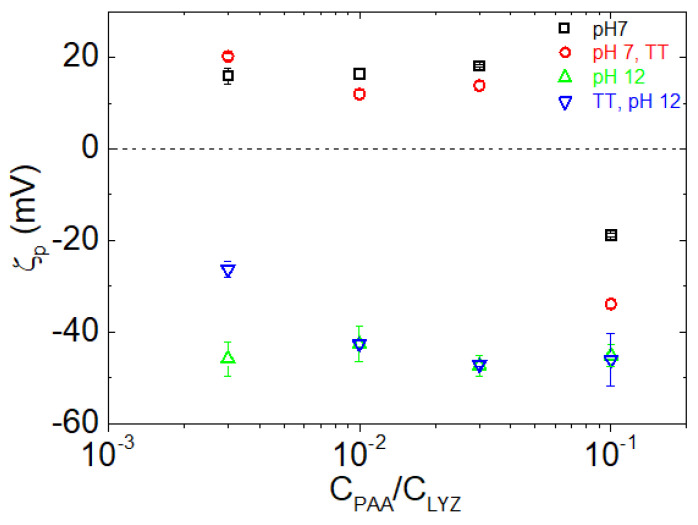
Zeta potential of PAA/LYZ NPs as a function of the C_PAA_/C_LYZ_ ratio under different conditions: pH 7 (black), TT at pH 7 (red), pH 12 (green), and TT at pH 12 (blue).

Zeta potential (ζ_p_) is a key indicator of the surface charge of NPs and thus the magnitude of electrostatic repulsion or attraction between particles. It is one of the most widely studied physicochemical parameters in nanotechnology, particularly due to its significant influence on the stability of dispersions and the interactions of nanocarriers with biological environments. The ζ_p_ values of PAA/LYZ NPs under different conditions are shown in Figure 4. At pH 7, there is no significant dependence on thermal treatment, and the complexes exhibit positive ζ_p_ values, except at the highest PAA content (r_m_ = 0.1), where the negative charge of the polyelectrolyte dominates, resulting in a net negative surface potential. This behavior is consistent with the fact that LYZ is positively charged under near-neutral conditions, thereby dictating the surface properties at low PAA concentrations. Thermal treatment causes minimal changes in ζ_p_, indicating that it has limited influence on surface charge or the interfacial architecture. However, at pH 12, ζ_p_ values become strongly negative across all mass ratios, reflecting the net negative charge of LYZ at pH > pI_LYZ_. TT samples at pH 12 maintain similar negative ζ_p_ values with the untreated ones.

The clear transition from positive to negative ζ_p_ with increasing PAA content and elevated pH highlights the tunable nature of the surface charge in these complexes. Such tunability is crucial for applications in which surface charge governs colloidal stability, bio-interactions, or transport properties.

FTIR and CD spectroscopy were employed to investigate LYZ conformation and secondary structural changes within PAA/LYZ complexes. These two techniques offer complementary insights. FTIR detects characteristic bond vibrations associated with specific secondary structure elements. Circular dichroism is highly sensitive to the chiral environment of protein structures and is widely used to monitor conformational transitions [1,30].

**Figure 5:**
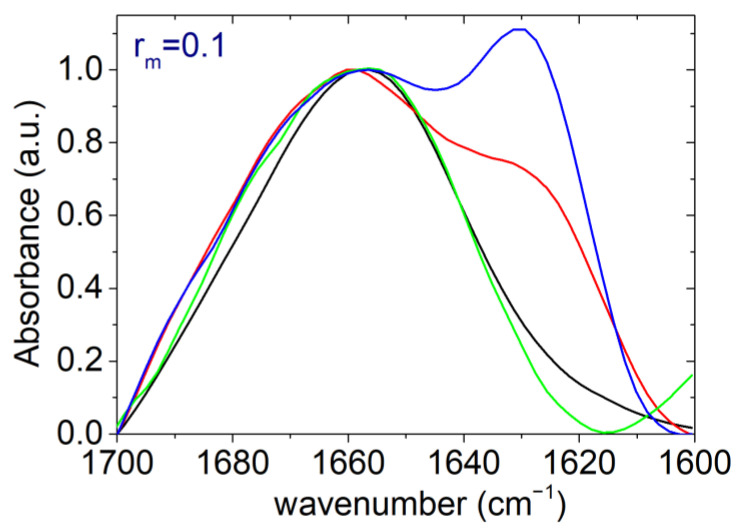
FTIR absorbance spectra at the Amide I region (1700–1600 cm^−1^) for complexes with mass ratio r_m_ = 0.1 at pH 7 (black), thermally treated at pH 7 (red), at pH 12 (green), and at pH 12 after thermal treatment at pH 7 (blue).

In this context, Figure 5 presents the FTIR spectra of the PAA/LYZ complexes with the highest PAA content (r_m_ = 0.1). What clearly stands out is the appearance of a new and more intense absorption band around ~1630 cm^−1^, which is indicative of increased *β*-sheet content. This band does not appear in the spectra of native LYZ under any pH condition or after thermal treatment (see S.I. Figure S2). This assignment is consistent with multiple literature sources that attribute absorption in the 1615–1635 cm^−1^ region to *β*-sheet structures in proteins [52,81,82,83,84,85]. The full absorbance spectra of LYZ, as well as the Amide I region for both LYZ and PAA/LYZ complexes under all tested conditions, are presented in Figure S2.

Regarding the CD results, an extensive analysis of the secondary structure of LYZ and complexes (r_m_ = 0.01 and 0.03) at pH 7 has already been conducted in a previous study by our group [52]. In the present work, we focus on the effect of an alkaline environment (pH 12) and the impact of thermal treatment on the structural integrity of the protein under these conditions. Experimental results show that PAA does not alter LYZ’s secondary structure at pH 12. In contrast, thermal treatment leads to a complete loss of *α*-helix content, indicating significant structural disruption. These findings were confirmed by repeat CD (Figure S3) and UV–Vis measurements (Figure S4). The contributions to the secondary structure of LYZ, as extracted from CD measurements in both the free state and in complexes, are presented in Figure S5.

In summary, the formation of PAA-LYZ nanoparticles at pH 7 was primarily led by the attractive electrostatic interaction between the negatively charged PAA and the positively charged LYZ. At pH 12, the destabilization of the NPs occurred, and it was hypothesized that this is because electrostatic interaction becomes overall repulsive. When the NPs were TT at pH 7, the destabilization of the NPs at pH 12 was prevented. This was attributed to the thermally induced denaturation at elevated temperature and the formation of protein–protein hydrophobic contacts upon cooling. This behavior of the protein, which has been established in pure protein solutions [35], motivated the development of this stabilization protocol to several protein–ionic polysaccharide systems. The experimental work proves the stabilization of the NPs upon thermal treatment at pH < pI_LYZ_, against disintegration at pH > pI_LYZ_. This is very important for industrial applications as in food science and medicine.

However, the experiments afford limited insight into the underlying mechanisms, primarily through measurements of the protein’s structural parameter transitions, which suggest protein–protein contacts form upon cooling. On the other hand, the atomistic molecular dynamics simulations provide information for the way that the thermal process affects the network formation. The microscopic mechanism of complexation is analyzed and quantified at the atomic level. The binding between pairs of the components (i.e., LYZ-LYZ, PAA-LYZ, and PAA-PAA) is quantified, highlighting the major driving forces of association and analyzing different types of energetic interactions (i.e., electrostatic, Van der Waals), hydrogen bonding, and conformational changes. The thermal process is simulated at both pH 7 and pH 12, providing crucial understanding of molecular associations at different conditions.

### 3.2. Simulation Results

In the following, results derived from atomistic simulations are presented, including a detailed analysis of energetics, association rates, and conformational representations. While SLS/DLS/ELS experiments correspond to macroscopic and completely formed aggregates, the atomistic simulations reveal the microscopic origins of their behavior by capturing the initial stages of LYZ-PAA complexation at the nanometer scale. The disintegration of NPs observed in SLS/DLS at pH 12 aligns with the reduced electrostatic and van der Waals interactions calculated in the simulations. The stability of thermally treated NPs at high pH, as shown by SLS/DLS, is also reflected in the simulations, which reveal increased polymer–polymer association and persistent LYZ-PAA contacts after quenching. Structural changes detected by FTIR and CD, including partial unfolding and rearrangement, are supported by the conformational shifts and new residue-level interactions captured in the simulations. Furthermore, changes in zeta potential across pH values correspond to the pH-dependent redistribution of charged residues observed computationally (association rates). The complementarity of the two approaches reveals the molecular mechanisms behind the experimentally observed stabilization of the PAA-LYZ complexes upon thermal treatment.

#### 3.2.1. Post-Simulation Snapshots

**Figure 6:**
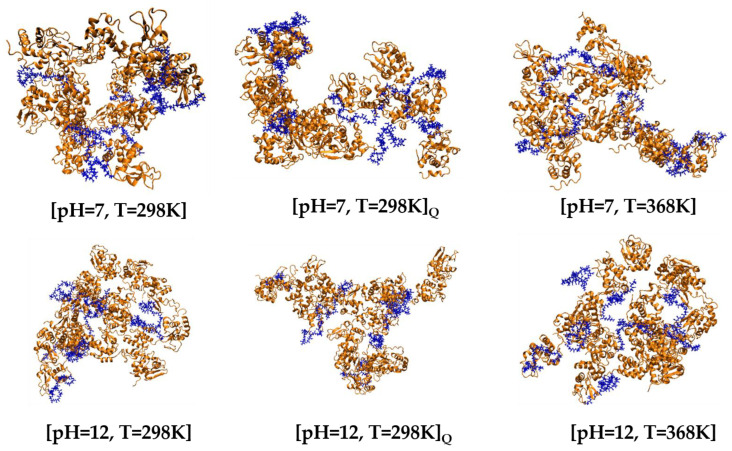
Representative post-simulation snapshots of [LYZ-PAA] complexes at different pH levels and temperatures. Q stands for the quenching run.

The network formation of LYZ molecules with PAA polymer chains is presented in the characteristic snapshot of Figure 6, where conformations of the model systems are juxtaposed at ambient conditions (T = 298 K), at high temperature (T = 368 K) and at T = 298 K after quenching, at both pH = 7 and pH = 12 conditions. Networks are observed in all cases with different conformational characteristics, where polymer chains form bridges among protein molecules. The symbol Q is used to denote the quenched system (i.e., after cooling from 368 K to 298 K).

In the following, an energetic analysis in terms of binding energies and hydrogen bonding is presented, revealing the mechanisms of the networks’ formation and stabilization.

#### 3.2.2. Energetic Calculations 

**Figure 7:**
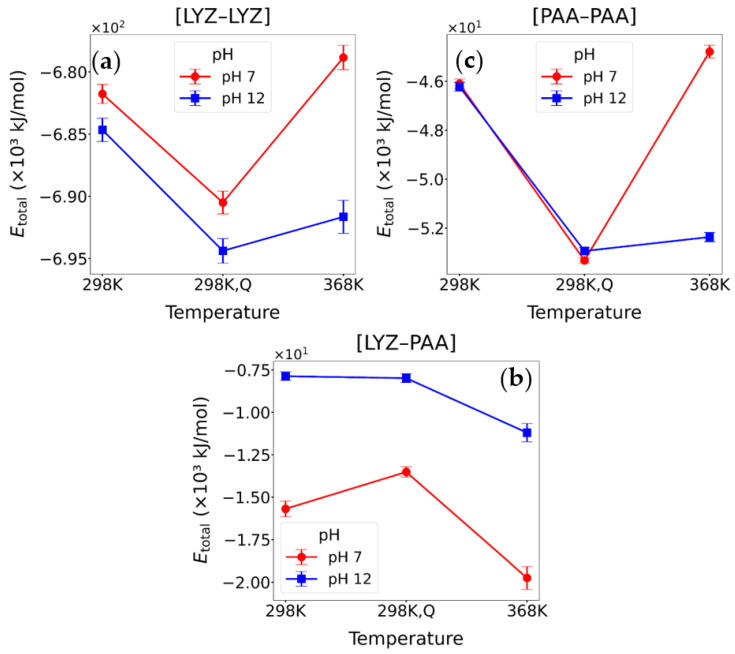
Total interaction energy (E_Total_ in kJ/mol) for different components of the [LYZ-PAA] complex under varying pH and temperature conditions. (**a**) E_Total_ between LYZ-LYZ molecules, (**b**) E_Total_ between LYZ-PAA molecules, and (**c**) E_Total_ between PAA-PAA molecules; values are averaged over the last 100 ns of simulation.

In order to gain a more complete understanding of the formation and stability of the [LYZ-PAA] complexes under different pH and temperature conditions, we calculated the thermodynamic properties and energetics of the systems. Specifically, we evaluated the solvation energy (ΔE_solv_), which captures the effect of solvent reorganization upon complex formation, and the overall binding free energy (ΔG_binding_), which quantifies the net favorability of the complexation process. The results are presented in Table 2. We also calculated the van der Waals (E_LJ_), Coulombic (E_Coul_), and total interaction energies (E_Total_) between LYZ and PAA, excluding interactions with solvent molecules. Interaction energies were calculated at 298 K, 368 K, and after quenching back to 298 K at both pH values. The results for interaction energy and its components are presented in Figure 7, and corresponding values are shown in Table 3. All energy values were calculated from the last 100 ns, which corresponds to the equilibrated part of the trajectory.

Additional information for the effect of the environmental factors on the complex formation and stability is provided through the calculation of the binding free energy for the [LYZ-PAA] complexes, highlighting the role of the solvation energy. The results are presented in Table 2 for T = 298 K, T = 368 K, and after quenching back to T = 298 K, at both pH values. All energy values were calculated from the last 100 ns, which correspond to the equilibrated part of the trajectory.

At pH 7, binding is favorable (negative ΔG_binding_) in all states, but after quenching, the affinity weakens slightly, compared to the high-temperature (368 K) system, pointing to a partially irreversible process. At pH 12, in the quenched system, ΔG_binding_ remains positive (repulsive) and higher than in the initial 298 K system, reinforcing the view that thermal disruption at basic pH leads to a persistent loss of favorable interactions and reflects an irreversible alteration in the binding energy. Notably, ΔE_solv_ underlines the critical role of hydration effects in modulating complex stability. At pH 7, ΔE_solv_ is strongly positive, indicating that significant energy is associated with reorganizing the solvent environment, as water molecules are displaced from the protein and polymer surfaces during complex formation. This is effectively compensated by the favorable Coulombic and van der Waals interactions, leading to a stable complex. At pH 12, however, ΔE_solv_ becomes markedly negative, reflecting an environment where the hydration of the separate protein and polymer species is energetically more favorable, particularly due to increased exposure of hydrophilic regions and altered solvent structuring, resulting from the conformational changes (e.g., emergence of β-sheets). This profound shift in hydration energy with pH and temperature plays a key role in determining the overall binding free energy.

In Figure 7a, the [LYZ-LYZ] total interaction energies become more negative at pH 12 than at pH 7 at all thermal states. This suggests stronger protein–protein attraction under basic conditions, likely due to a rearrangement of charged residues and the formation of new inter-protein contacts. Moreover, after quenching, the attraction is stronger compared to both low- and high-temperature systems. For [LYZ-PAA] interactions (Figure 7b), the high pH leads to weaker attractions at all thermal states. E_Total_ decreases after quenching, compared to both low- and high-temperature systems, with a more pronounced difference at pH 7, whereas at pH 12, the system almost restores the initial interaction strength. In contrast, [PAA-PAA] interactions (Figure 7c) are almost equal at the low temperature and the corresponding state after quenching (298 K), whereas a considerable difference is observed at the high temperature (368 K), indicating much stronger PAA-PAA attraction under basic conditions. In addition, the quenching process points to an almost irreversible change at pH 12, while at pH 7, it leads to E_Total_ values being more negative than the ones in both low- and high-temperature systems.

In the following, the individual energetic components are presented. As shown in Table 3, the comparison of the two pH conditions reveals that van der Waals interactions between LYZ and PAA are consistently more favorable (i.e., stronger attraction) at pH 7 than at pH 12, under all thermal states. Therefore, at 298 K, E_Total_ at pH 7 is considerably more negative than at pH 12, indicating stronger interactions under acidic conditions. A similar difference is observed at 368 K, whereas after the quenching, E_LJ_ does not attain the same values as the ones at T = 298 K. This indicates an irreversible process, which at pH 7 leads to an increased E_LJ_ value, compared to the initial temperature of quenching (368 K), but decreased compared to the one that corresponds to the 298 K system (i.e., partial reversibility). However, at pH 12, the Van der Waals interactions are completely irreversible, within statistical uncertainties, after quenching. In contrast, electrostatic interactions behave quite differently. At pH 7, E_Coul_ remains strongly negative under all thermal conditions, indicating persistent electrostatic attraction between LYZ and PAA. Moreover, quenching causes a partial reversible change. At pH 12, considerable attenuation of attraction is observed at all three thermal states. Interestingly, the quenching process results in weaker electrostatic attractions between the protein and polyelectrolyte, compared to the corresponding systems at 298 K as well as to the initial temperature of quenching (368 K) at both pH values.

However, different inter-protein and inter-polymer associations are created and destroyed after the quenching process, establishing a stable network. Therefore, no big differences are observed in the interactions between LYZ molecules, but rather small conformational changes, partially irreversible, reflected in the E_LJ_, at both pH values. On the other hand, enhanced electrostatic attractions appear among PAA chains, after the thermal treatment, which at pH 7 are stronger than the ones at the initial quenching temperature and those in the corresponding system at the same temperature. At pH 12, electrostatics are irreversible after thermal treatment, retaining the higher E_Coul_, value which is realized at T = 368 K. Smaller differences in the Van der Waals interactions show a reduced and irreversible attraction at pH 7, whereas reversibility in E_LJ_ is observed at pH 12 after quenching.

These findings suggest that enhanced polymer–polymer interactions within the network play a vital role in stabilizing the complex, as evidenced by Figure 1, Figure 2 and Figure 3 and discussed in Section 3.1. Thermally treated samples at pH 12 maintain their integrity, whereas untreated samples at pH 12 are greatly compromised.

Table 4 presents the average number of hydrogen bonds between all pairs of components in the systems, offering further insight into how the thermal treatment at different pH levels affects network formation and stability. At pH 7, an elevated temperature to 368 K weakens both inter-protein and protein–water hydrogen bonds while strengthening LYZ-PAA hydrogen bonds. Upon quenching the system back to 298 K, all hydrogen bonds rebound nearly to their original values, indicating that the proximity among proteins and their hydration recovered. Similarly, a reversible reduction in hydrogen bonding among PAA and water molecules is observed, whereas PAA-PAA hydrogen bonding is not affected by the temperature. Analogous is the behavior at pH 12, indicating a high degree of reversibility in the amount of hydrogen bonds formed at basic pH. Slight differences are observed at T = 368 K, where a small disruption of HBs between LYZ molecules is found, with a corresponding enhancement in polymer hydrogen bonds, compared to the low-temperature conditions. Interestingly, the [LYZ-PAA] complex hydrates similarly at 368 K under both pH conditions, as evidenced by the [LYZ-W] and [PAA-W] hydrogen bonds. On the other hand, at 298 K, different hydration is observed between the two pH levels with the one at pH 12 being higher. Comparing [LYZ-W] and [PAA-W] hydrogen bonds, the difference is detected in protein’s exposure to water, while for polyelectrolyte chains, hydrogen bonding is almost equal. This reflects conformational changes in protein molecules at basic pH, which are analyzed in detail in the next subsection. After quenching, the hydrogen bonds with water revert to their original amount. This suggests that hydrogen bonding is largely reversible after thermal treatment, and this effect is consistent across both pH values. The reformation of hydrogen bonds and recovery of hydration shells observed in the simulations, leading to a different hydrogen bond pattern after quenching, likely contributes to maintaining the integrity of nanoparticles under basic conditions that would otherwise promote disintegration.

The combination of hydrogen bond analysis with energetic calculations highlights that quenching results in reduced electrostatic attractions between proteins and polyelectrolytes, while Van der Waals interactions are enhanced. In addition, it induces increased associations between polymer chains in the network, which significantly contribute to complex stabilization. The amount of hydrogen bonds is almost the same after the thermal treatment; however, there are differences in the hydrogen bond network, since partially irreversible changes in the energy components are observed, which indicate conformational variations. As a result, the recovery of the initial binding pattern is prevented.

#### 3.2.3. Association Rates

**Figure 8:**
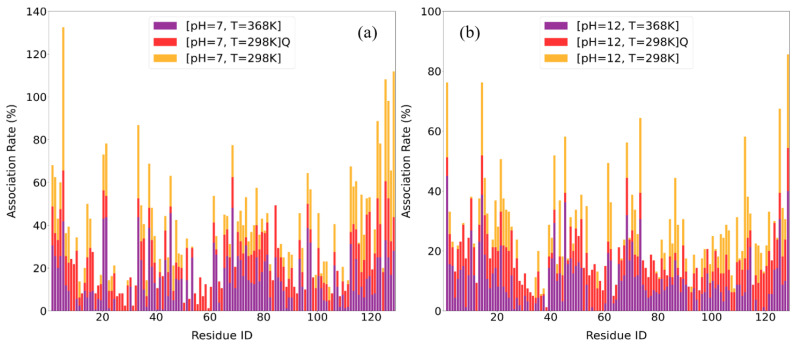
Comparison of residue association rates of LYZ with PAA at different temperatures: (**a**) pH = 7 and (**b**) pH = 12, represented as histograms calculated from the last 100 ns of the trajectory for all (129) residues.

To further elucidate the molecular origins of binding affinity at specific conditions, we calculated the residue-specific association rates between LYZ and PAA across different pH and temperature conditions. The calculation method of association rates is described in detail in our previous paper [53]. Figure 8 presents the association rates for all 129 residues, while Table 5 lists the top 10 most highly associated residues. These rates, derived from spatial proximity metrics over time, reveal how specific amino acids contribute to the interaction landscape and how their behavior reflects broader thermodynamic and hydrogen bonding trends [53].

A partially reversible behavior is obvious at both pH values (Figure 8a,b) after the quenching process. At pH 7, the strongest binders at 298 K are dominated by hydrophilic, cationic residues (ARG5, ARG125, ARG128, LYS33), in line with the highly favorable electrostatic energies. Quenching from 368 K back to 298 K nearly fully restores the amount of the formed hydrogen bonds between proteins (i.e., [LYZ-LYZ]) and the hydration shell of LYZ (i.e., [LYZ-W] Hbs). Despite this, the ranking of residues concerning LYZ-PAA association fails—neutral hydrophilic side chains (ASN113, THR118, ASP119) enter the top ten—showing that quenching does not fully reestablish the pre-heated electrostatic interface. Likewise, heat-activated hydrophilic contacts (ARG21, ARG45, ARG68, LYS96) that appear only at 368 K are not retained after quenching. These findings also claimed the partial reversibility of LYZ-PAA complex with thermal treatment (i.e., ARG125 and ARG5 remain at the top 10 associations, after quenching, at acidic pH). Although the protein fold and hydration shell recover, the residue-level binding map remains reconfigured, with contributions from both low-T and heat-promoted hydrophilic interactions.

By contrast, at pH 12, the 298 K association profile is weaker and still led by hydrophilic, cationic residues (ARG112, ARG125, ARG128, LYS1). After quenching, none of these charged residues regain their initial prominence; instead, hydrophobic residues (LEU8, LEU56, LEU129, TRP123, TYR23) dominate the top ten. Although the overall hydrogen bond counts recover, the residue-level association map is irreversibly rerouted toward non-electrostatic, hydrophobic contacts. Even transient charged contacts seen at 368 K (ARG14, ARG45, ARG68, ARG125) do not persist post-quench. Thus, under alkaline conditions, quenching regenerates the global H-bond amount but leaves the binding interface with PAA to be governed by hydrophobic interactions. As a result, more hydrophilic amino acids remain exposed to water and enhance the hydrogen bonding with water molecules. This indicates that thermal treatment favors the formation of NPs with a hydrophilic surface.

**Figure 9:**
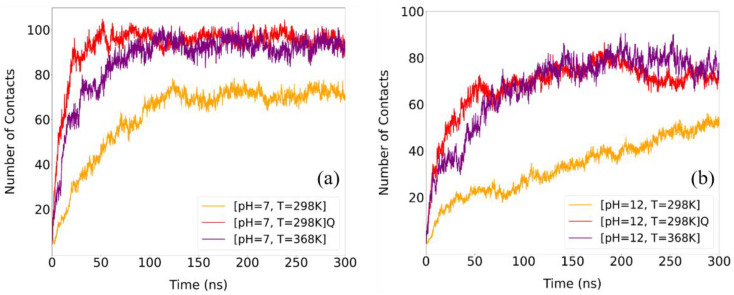
Time evolution of the number of contacts formed between alpha carbons of LYZ and PAA, for different temperatures and pH values equal to (**a**) pH = 7 and (**b**) pH = 12. Data are normalized with the total number of LYZ molecules and PAA chains.

Furthermore, the number of contacts between LYZ-LYZ and PAA has been calculated as a function of time based on the proximity of alpha carbon atoms between LYZ and PAA within a predefined distance, as described in our previous work [53]. A cutoff distance of 0.6 nm was selected, corresponding to approximately 1.5 times a Van der Waals diameter of typical heavy atom pairs (e.g., C–O) and accommodating thermal fluctuations observed in protein–polymer interactions. In Figure 9, the time evolution of total contacts between LYZ and PAA is presented. Enhanced contacts are observed at elevated temperature, whereas the total number of contacts remains high after quenching. This suggests that the overall association between protein and polymer is largely maintained, which supports the stabilization of the complexes.

Comparing the two pH conditions, the post-quenching contact profiles at both pH 7 and pH 12 closely resemble their corresponding high-temperature (368 K) states, indicating that the additional contacts formed at elevated temperature are largely preserved during cooling. At neither pH does the system recover the original 298 K contact levels, suggesting that the protein–polymer interface remains in a heat-stabilized configuration post-quenching. While this behavior is evident in both cases, the effect is more pronounced at pH 12, possibly reflecting a dominant role of hydrophobic interactions between parts of the molecules, which do not necessarily contribute to hydrogen bonds and are therefore less sensitive to temperature-induced structural changes.

The above findings align with experimental observations for the stabilization of the thermally treated samples. NP stability after thermal treatment lies on the irreversible or partially reversible properties of the LYZ-PAA formed network, in terms of local conformational changes, energetic interactions, and hydrogen bonding.

## 4. Conclusions

This work examines the thermal stabilization of LYZ-PAA NPs formed through electrostatic complexation. It is motivated by the increased interest in polyelectrolyte–protein complexation and the proven ability for stabilization when the thermally triggered protein aggregation is exploited. The experimental investigation confirms that PAA-LYZ complexes with a radius 100–200 nm are formed at pH 7 (pH < pI_LYZ_) and are disintegrated at pH 12 (pH > pI_LYZ_). When the thermal treatment protocol is applied at pH 7, the NPs resist the trend for disintegration at pH 12. The surface potential of the NPs is mostly dominated by the overall charge of LYZ, especially at pH 12. The changes in the secondary structure of LYZ, induced by thermal treatment, imply that LYZ-LYZ aggregation takes place within the NPs. These inter-protein associations have been motivated by the application of thermal treatment for the stabilization of protein–polysaccharide NPs.

However, experimental observations do not reveal the microscopic mechanism of complexation, especially during the initial phase of the process. The fundamental reasons for network formation and stabilization upon thermal treatment are thoroughly explored using atomistic molecular dynamics simulations. The effect of thermal treatment is explored at two pH conditions, alkaline (pH 7) and basic (pH 12), highlighting the combined role of conformational changes, energetic interactions, and hydrogen bonds on the stabilization of the formed network. Electrostatic interactions dominate under all pH and temperature conditions; however, thermal treatment has different effects on the individual energy components for each pair of molecules. Quenching is observed to weaken electrostatic attractions between proteins and polyelectrolytes but strengthen Van der Waals interactions. This process also enhances the association of polymer chains within the network, at both pH values, which is crucial for complex stabilization. Protein–protein attraction is stronger under basic conditions, whereas a limited effect of thermal treatment is observed on these interactions. Importantly, our thermodynamic analysis revealed that solvation energy plays a dominant role in governing complex stability, with significant changes in hydration upon thermal treatment and pH variation that directly modulate the free energy of binding.

Moreover, upon thermal treatment, hydrogen bonding does not make a significant quantitative contribution to complex stabilization; however, considerable changes in the hydrogen bond network take place, revealing a different binding pattern. As a result, the hydrophobicity of the NP surface changes, which can have potential applications in the binding/release of hydrophobic molecules with pH as a stimulus. In addition, elevated temperatures lead to enhanced number contacts between LYZ and PAA, which remain high after quenching. This implies that the protein and polymer largely stay associated, thus advocating for the stabilization of the complexes.

These findings support experimental observations regarding the stabilization of thermally treated samples, which stems from the irreversible or partially reversible nature of the LYZ-PAA network. The findings from this model system may be applied to other protein–polyelectrolyte systems for a systematic design of biocompatible prepared NPs.

## Figures and Tables

**Table 1:** Number of atoms for simulated LYZ-PAA systems at pH 7 and pH 12, including water, protein, and ions.

pH	Total Number of Atoms	Number of Water Atoms	Number of LYZ Atoms	Number of NA Atoms	Number of CL Atoms
7	403,211	368,343	31,360	402	370
12	403,282	368,286	31,296	594	370

**Table 2:** The solvation (ΔEsolv) and binding free energies (ΔGbinding) (kJ/mol) of the [LYZ-PAA] complexes at different pH and temperature conditions.

System	Condition	ΔE_solv_	ΔG_binding_
[LYZ-PAA]	pH = 7, T = 298 K	564,909.0 ± 269.2	−10,291.8 ± 7.9
[LYZ-PAA]Q	pH = 7, T = 298 K	500,421.5 ± 828.5	−9364.3 ± 19.3
[LYZ-PAA]	pH = 7, T = 368 K	576,917.5 ± 748.9	−11,161.2 ± 15.5
[LYZ-PAA]	pH = 12, T = 298 K	−189,394.4 ± 307.0	673.2 ± 5.4
[LYZ-PAA]Q	pH = 12, T = 298 K	−188,468.5 ± 381.4	854.7 ± 5.3
[LYZ-PAA]	pH = 12, T = 368 K	−178,359.4 ± 524.9	581.2 ± 9.2

**Table 3:** Lennard-Jones (E_LJ_), Coulombic (E_Coul_), and total interaction energies (E_Total_ in kJ/mol) between [LYZ-LYZ], [PAA-PAA], and [LYZ-PAA] at different pH and temperature conditions; Q stands for the quenching run.

System	Conditions	E_LJ_	E_Coul_	E_Total_
[LYZ-LYZ]	pH = 7, T = 298 K	−63,504.9 ± 233.9	−618,261.5 ± 774.8	−681,766.4 ± 775.2
[LYZ-LYZ]_Q_	pH = 7, T = 298 K	−61,795.4 ± 303.0	−628,704.3 ± 804.1	−690,499.7 ± 918.6
[LYZ-LYZ]	pH = 7, T = 368 K	−62,750.5 ± 259.5	−616,088.9 ± 970.3	−678,839.3 ± 979.1
[LYZ-LYZ]	pH = 12, T = 298 K	−60,626.1 ± 239.1	−624,033.2 ± 902.2	−684,659.1 ± 934.7
[LYZ-LYZ]_Q_	pH = 12, T = 298 K	−58,959.4 ± 271.7	−635,424.6 ± 914.4	−694,383.9 ± 986.8
[LYZ-LYZ]	pH = 12, T = 368 K	−56,963.7 ± 399.4	−634,682.5 ± 1206.5	−691,646.2 ± 1329.1
[LYZ-PAA]	pH = 7, T = 298 K	−2506.5 ± 95.5	−13,183.4 ± 442.7	−15,689.9 ± 463.3
[LYZ-PAA]_Q_	pH = 7, T = 298 K	−2989.4 ± 88.2	−10,526.1 ± 302.4	−13,515.5 ± 299.5
[LYZ-PAA]	pH = 7, T = 368 K	−3295.8 ± 118.8	−16,453.1 ± 653.8	−19,748.9 ± 674.4
[LYZ-PAA]	pH = 12, T = 298 K	−1517.8 ± 116.1	−6351.9 ± 241.1	−7869.7 ± 251.9
[LYZ-PAA]_Q_	pH = 12, T = 298 K	−2253.9 ± 83.8	−5731.5 ± 255.2	−7985.5 ± 260.5
[LYZ-PAA]	pH = 12, T = 368 K	−2535.5 ± 116.4	−8665.1 ± 514.8	−11,200.6 ± 541.3
[PAA-PAA]	pH = 7, T = 298 K	−2325.1 ± 48.1	−43,775.7 ± 190.7	−46,100.7 ± 190.2
[PAA-PAA]_Q_	pH = 7, T = 298 K	−2184.6 ± 55.8	−51,141.2 ± 99.3	−53,325.8 ± 107.1
[PAA-PAA]	pH = 7, T = 368 K	−2154.1 ± 61.8	−42,635.4 ± 257.5	−44,789.5 ± 260.1
[PAA-PAA]	pH = 12, T = 298 K	−2494.9 ± 64.9	−43,724.1 ± 198.1	−46,218.9 ± 183.8
[PAA-PAA]_Q_	pH = 12, T = 298 K	−2422.3 ± 55.5	−50,515.9 ± 125.9	−52,938.3 ± 129.7
[PAA-PAA]	pH = 12, T = 368 K	−2638.5 ± 73.4	−49,728.6 ± 180.3	−52,367.2 ± 189.2

**Table 4:** Average number of hydrogen bonds (HBs) formed between LYZ and LYZ ([LYZ-LYZ]), LYZ and water ([LYZ-W]), LYZ and PAA ([LYZ-PAA]), shown per LYZ molecule, PAA and PAA ([PAA-PAA]), and PAA and water ([PAA-W]), shown per PAA polymer chain, during the last 100 ns of the simulation.

Condition	[LYZ-LYZ]	[LYZ-W]	[LYZ-PAA]	[PAA-PAA]	[PAA-W]
[pH = 7, T = 298 K]	102.5 ± 1.2	259.7 ± 2.2	11.6 ± 0.5	1.4 ± 0.3	108.1 ± 2.4
[pH = 7, T = 298 K]_Q_	101.2 ± 1.2	259.2 ± 2.2	12.2 ± 0.5	1.3 ± 0.3	105.5 ± 2.2
[pH = 7, T = 368 K]	98.1 ± 1.3	231.1 ± 2.4	14.1 ± 0.7	1.3 ± 0.3	86.8 ± 2.5
[pH = 12, T = 298 K]	99.2 ± 1.2	268.6 ± 2.4	7.6 ± 0.4	1.4 ± 0.3	109.5 ± 2.6
[pH = 12, T = 298 K]_Q_	98.6 ± 1.3	268.2 ± 2.7	7.4 ± 0.4	1.5 ± 0.3	104.5 ± 2.7
[pH = 12, T = 368 K]	95.8 ± 1.6	232.3 ± 3.2	10.1 ± 0.6	1.8 ± 0.4	81.5 ± 2.7

**Table 5:** Top 10 residues with highest association rates across all different pH and temperature conditions.

Condition	Top 10 Residues with Highest Association Rates
[pH = 7, T = 298 K]	ARG5, ARG128, ARG125, TRP123, GLY126, LYS33, ALA122, CYS127, ARG112, LYS116
[pH = 7, T = 298 K]_Q_	ASN113, THR118, ASP119, ALA122, ARG125, GLY126, LEU129, LEU8, LEU84, ARG5
[pH = 7, T = 368 K]	ARG21, ARG45, ARG68, ARG5, TYR20, LYS33, LYS96, ASN37, ARG125, LYS1
[pH = 12, T = 298 K]	ARG112, ARG128, LYS1, ARG14, ARG61, SER86, ARG125, ASN19, ARG21, GLN41
[pH = 12, T = 298 K]_Q_	LEU 129, LEU8, THR51, SER24, LEU56, TRP123, GLY16, TYR23, LEU25, VAL120
[pH = 12, T = 368 K]	LYS1, ARG14, ARG45, ARG128, ARG68, ARG125, ALA10, LYS13, GLN41, ARG73

## Data Availability

The original contributions presented in this study are included in the article/Supplementary Material. Further inquiries can be directed to the corresponding authors.

## Supplementary Materials

The following supporting information can be downloaded at https://www.mdpi.com/article/10.3390/polym17152125/s1: Figure S1: Hydrodynamic radius distribution for all mass ratios at pH 7 (a) and for all applied treatments for rm = 0.03 (b) and rm = 0.1 (c). Figure S2: FTIR absorbance spectra of LYZ and PAA/LYZ complexes. (a) Full-range spectra (500–3500 cm^−1^) of LYZ at pH 7 (black), TT at pH 7 (red), at pH 12 (green), and at pH 12 after TT at pH 7 (blue). The inset shows the spectrum of NaOH (orange). (b and c) Amide I region (1700–1600 cm^−1^) for complexes with mass ratios r_m_ = 0.03 and 0.1, respectively, revealing pH- and temperature-induced shifts associated with changes in secondary structure. Figure S3: CD spectra of LYZ (red) and PAA/LYZ complexes at pH 12 with representative rm 0.01 (blue) and 0.03 (green), (a) without thermal treatment and (b) upon thermal treatment. Figure S4: UV–Vis absorbance spectra of LYZ solutions at various concentrations (a) and corresponding calibration curve at 280 nm (b). Figure S5: Contributions to secondary structure of LYZ extracted from CD results in free state (a) and in complexes with r_m_=0.01 (b) and r_m_=0.03 (c). References [86,87] are cited in the supplementary materials.
