# Supplementary material for: Complexation and Thermal Stabilization of Protein–Polyelectrolyte Systems via Experiments and Molecular Simulations: The Poly(acrylic acid)/Lysozyme Case
Source: Polymers (Basel). 2025 Aug 1;17(15):2125. doi: 10.3390/polym17152125 (PMC12349267; doi:10.3390/polym17152125)
Supplement: Supplementary file 1 [file polymers-17-02125-s001.zip › polymers-3722998-supplementary.pdf]

Supporting Information

Complexation and Thermal Stabilization of Protein–Polyelectrolyte Systems via Experiments and Molecular Simulations: The Poly(acrylic acid) / Lysozyme Case

Sokratis N. Tegopoulos[1], Sisem Ektirici[2], Vagelis Harmandaris[2,3,4], Apostolos Kyritsis[1], Anastassia N. Rissanou[5] and Aristeidis Papagiannopoulos[5]

[1] *School of Applied Mathematical and Physical Sciences, National Technical University of Athens, GR-15772 Athens, Greece*

[2] *Computation-Based Science and Technology Research Center, The Cyprus Institute, Nicosia 2121, Cyprus*

[3] *Department of Mathematics and Applied Mathematics, University of Crete, GR-71409 Heraklion, Greece*

[4] *Institute of Applied and Computational Mathematics, Foundation for Research and Technology Hellas, IACM/FORTH, GR-71110 Heraklion, Greece*

[5] *Theoretical & Physical Chemistry Institute, National Hellenic Research Foundation, 48 Vassileos Constantinou Avenue, GR-11635 Athens, Greece*

** Corresponding authors: apapagiannopoulos@eie.gr, trissanou@eie.gr*

Size distributions

At mass ratios r_m = 0.03 and 0.1, the formation of PAA-LYZ nanocomplexes at pH 7 of a well-defined size distribution is evident (Figure S1a). At r_m = 0.03, strong secondary aggregation is found after thermal treatment (Figure S1b). Nevertheless, at r_m = 0.1, thermal treatment does not change the size distribution significantly and the NPs are stable at pH 12, contrary to the case of no thermal treatment (as discussed in the main text).

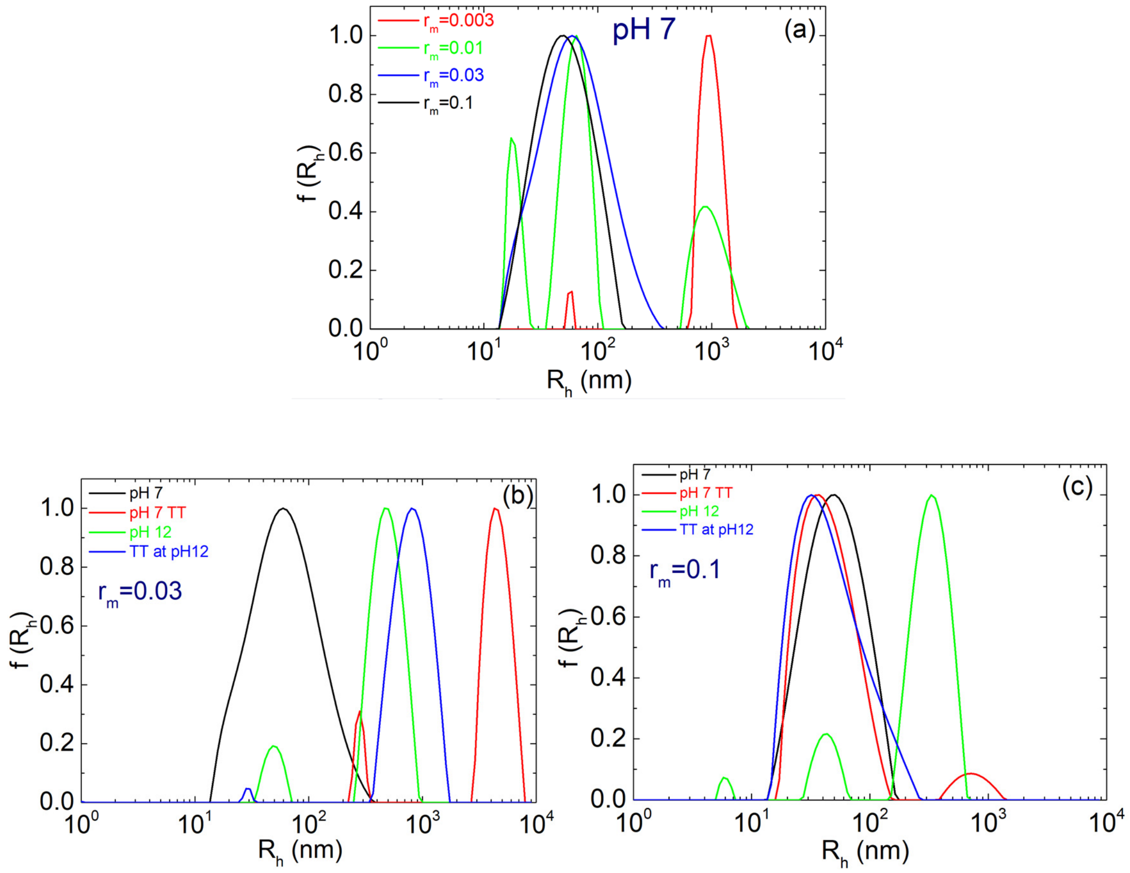

Figure S1. Hydrodynamic radius distribution for all mass ratios at pH 7 (a) and for all applied treatments for r_m = 0.03 (b) and r_m = 0.1 (c).

FTIR experiments

Figure S2a presents the FTIR spectra of LYZ in the range of 500–3500 cm^{-1}. The full spectral range is shown to highlight the existence of two distinct peaks at approximately 1564 and 1433 cm^{-1}, which appear only in the samples at pH 12. The inset of the same figure magnifies this region and includes the spectrum of pure NaOH for comparison. The clear overlap of these peaks with those in the NaOH spectrum confirms their assignment to residual NaOH used to adjust the pH from 7 to 12. Another important finding (Figure S2b) is that the main peak (~1658 cm^{-1} at pH 7) shifts to lower wavenumbers (1645–1635 cm^{-1}) when the pH is increased to 12, both with and without thermal treatment. This shift indicates an enhancement in β-sheet content [1,2]. Analysis of the Amide I region (1700–1600 cm^{-1}) at pH 7, which is sensitive to the

backbone conformations of proteins, was previously conducted in our earlier work [3]. That study demonstrated that thermal treatment at pH 7 leads to a notable increase in absorbance in the 1600–1630 cm^{-1} range, typically associated with β-sheet structures. This finding supports the hypothesis that thermal treatment induces partial unfolding and structural reorganization of LYZ, promoting the formation of intermolecular β-sheet aggregates, and appears at both pH 7 and 12.

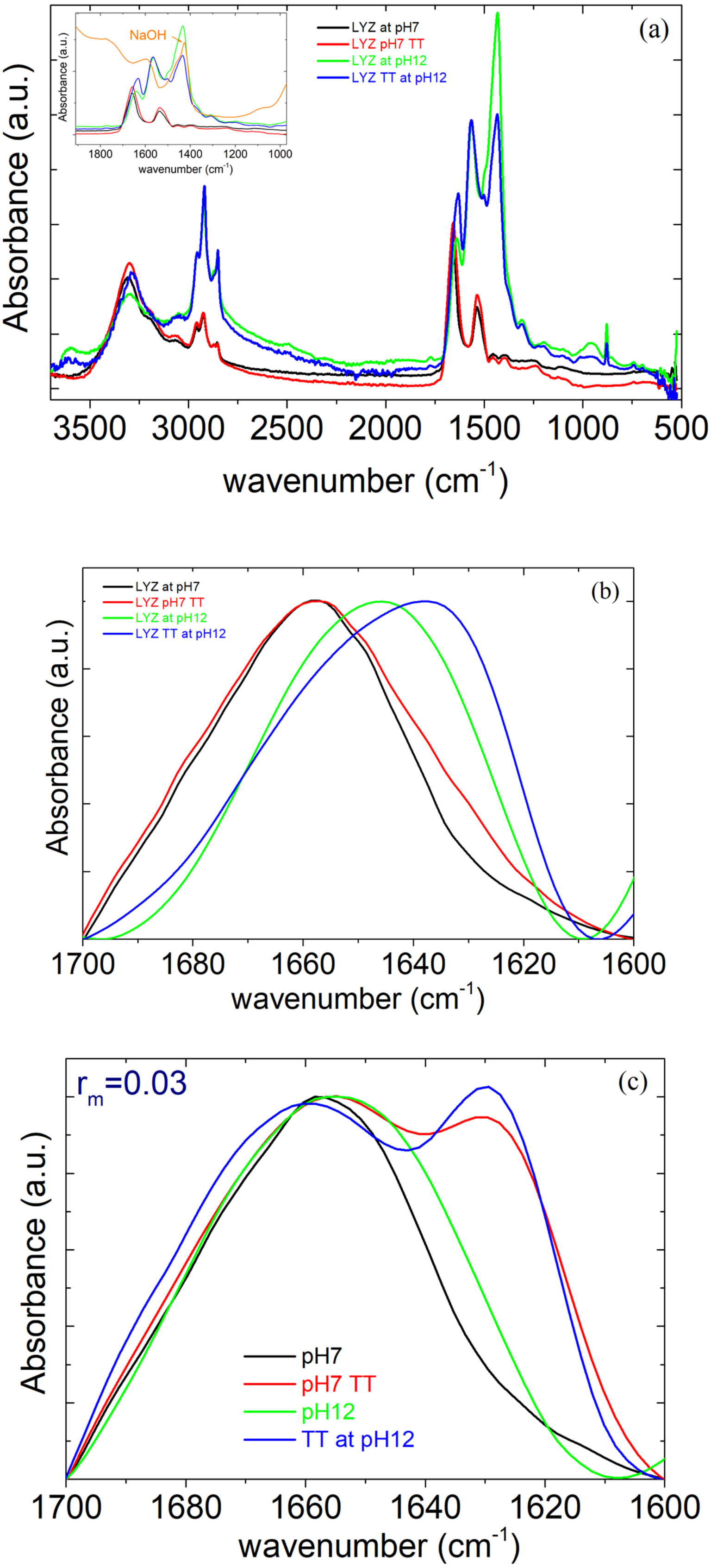
(a)
Absorbance (a.u.)
wavenumber (cm-1)
3500
3000
2500
2000
1500
1000
500
LYZ at pH7
LYZ pH7 TT
LYZ at pH12
LYZ TT at pH12
NaOH
Absorbance (a.u.)
1800
1600
1400
1200
1000
wavenumber (cm-1)
(b)
Absorbance (a.u.)
LYZ at pH7
LYZ pH7 TT
LYZ at pH12
LYZ TT at pH12
1700
1680
1660
1640
1620
1600
wavenumber (cm-1)
(c)
r_m=0.03
Absorbance (a.u.)
pH7
pH7 TT
pH12
TT at pH12
1700
1680
1660
1640
1620
1600
wavenumber (cm-1)

Figure S2. FTIR absorbance spectra of LYZ and PAA/LYZ complexes. (a) Full-range spectra (500–3500 cm^{-1}) of LYZ at pH 7 (black), TT at pH 7 (red), at pH 12 (green), and at pH 12 after TT at pH 7(blue). The inset shows the spectrum of NaOH (orange). (b and c) Amide I region (1700–1600 cm^{-1}) for complexes with mass ratios r_m = 0.03 and 0.1, respectively, revealing pH- and temperature-induced shifts associated with changes in secondary structure.

Figure S2c shows the Amide I region of the FTIR spectra for the PAA/LYZ complexes at a representative molar ratio of r_m = 0.03. A distinct and more intense absorption band emerges ~1630 cm^{-1}, which is indicative of increased β-sheet content in the complexes. This band is notably absent from the spectra of thermally treated LYZ (Figure S2b). Possibly, the higher concentration of LYZ within the NPs in comparison to free LYZ allows for more effective inter-protein β-sheet formation.

CD experiments

As shown in Figure S3a, the presence of PAA at pH 12 does not significantly affect the secondary structure of LYZ, as the CD spectra of the free protein and the complexes at both mass ratios are nearly identical. In contrast, Figure S3b highlights that the α-helix structure is completely lost after thermal treatment at r_m=0.03.

To confirm this remarkable result, the CD measurements were repeated under identical conditions. Furthermore, to ensure that LYZ was still present in the tested samples, UV–Vis spectrophotometry was performed on the stock LYZ solution used in the sample preparation (Figure S4). The characteristic absorbance peak at λmax = 280 nm, attributed to aromatic residues in the protein, was used to monitor LYZ concentration. This calibration curve was employed to verify that LYZ remained present in the tested samples after complexation and

treatment, ensuring that the observed structural changes were not due to protein degradation or loss. The protein concentration was confirmed to be within the expected range at all conditions, thereby excluding any possibility of protein phase separation from solution.

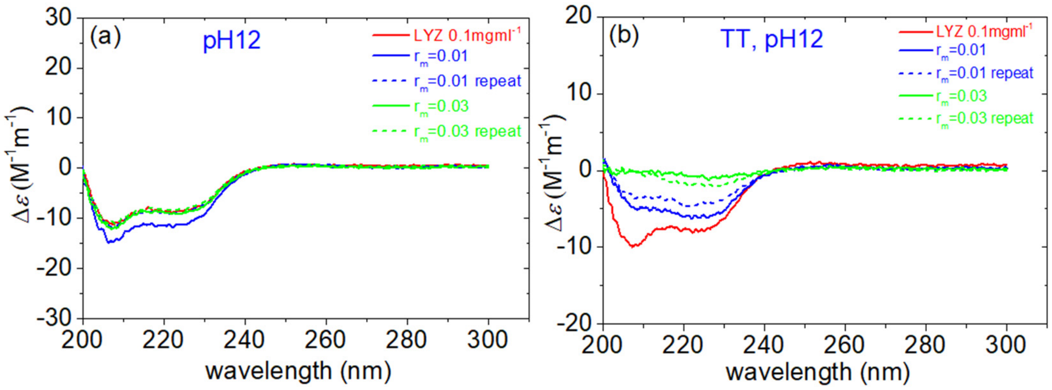

Figure S3. CD spectra of LYZ (red) and PAA/LYZ complexes at pH 12 with representative r_m 0.01 (blue) and 0.03 (green), (a) without thermal treatment and (b) upon thermal treatment.

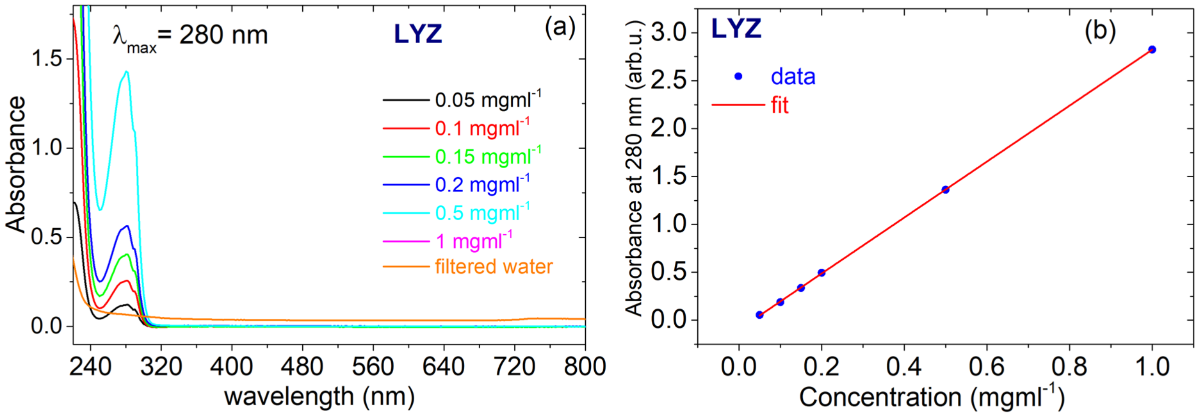

Figure S4. UV–Vis absorbance spectra of LYZ solutions at various concentrations (a) and corresponding calibration curve at 280 nm (b).

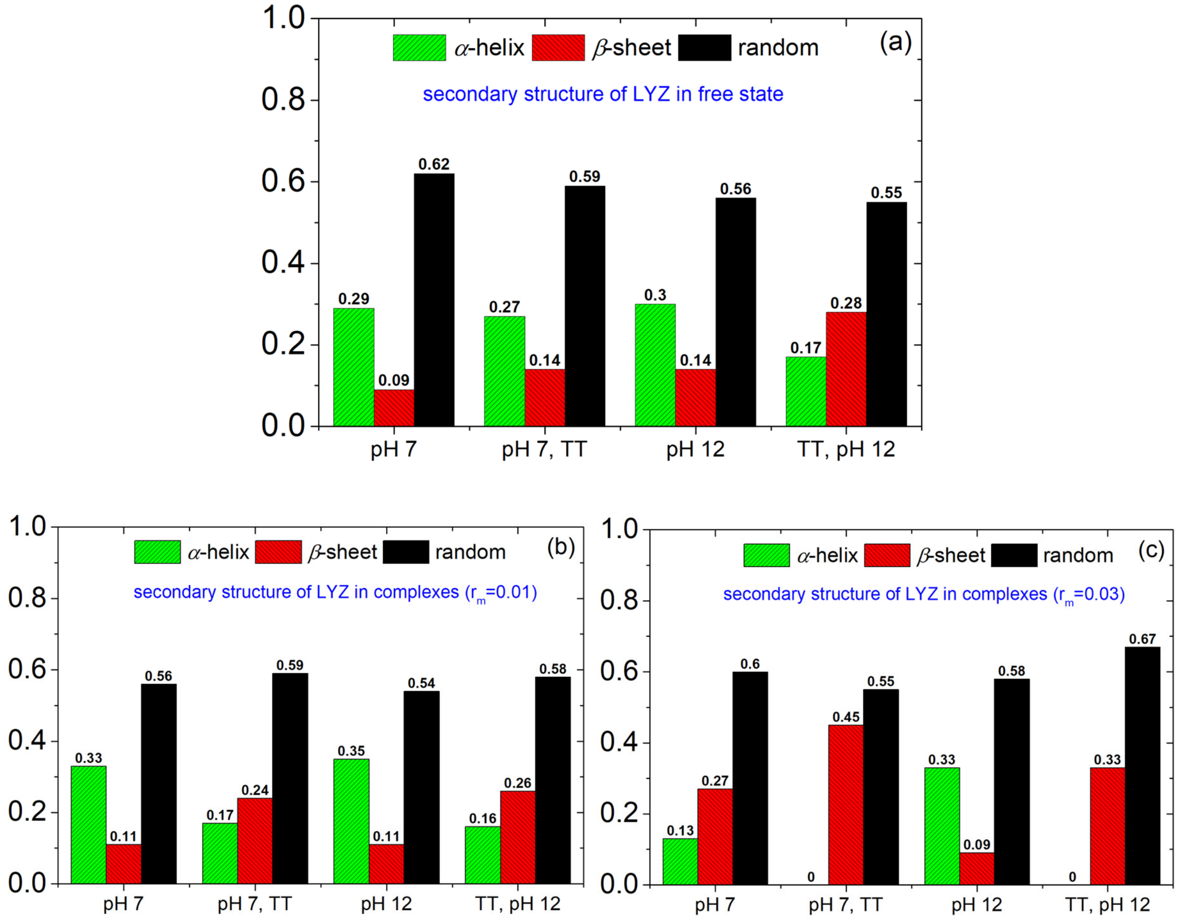

Figure S5. Contributions to secondary structure of LYZ extracted from CD results in free state (a) and in complexes with r_m=0.01 (b) and r_m=0.03 (c).

Figure S5 quantifies the observations from CD measurements, presenting the contribution of different secondary structures. For completeness, the results at pH 7 from our recent work [3] are also included. Figure S5 shows the secondary structure composition of free LYZ under all tested conditions. At pH 7, following thermal treatment, the α-helix content decreases slightly from 29% to 27%, accompanied by an increase in β-sheet content from 9% to 14%, while the random coil fraction remains nearly unchanged. At pH 12 without thermal treatment, the secondary structure distribution remains like the one at pH 7. However, a significant structural change is observed when thermal treatment is applied. In this case, α-helix content drops sharply from 30% to 17%, while β-sheet content increases from 14% to 28%. This shift is consistent with the FTIR results, supporting the hypothesis of enhanced β-sheet formation and

partial unfolding under combined thermal and alkaline stress. For the PAA/LYZ complexes (Figure S5b and S5c), thermal treatment induces a pronounced loss of α-helix accompanied by a concomitant increase in β-sheet structure. Notably, at r_m=0.03, thermal treatment entirely overcomes the α-helix signal, and this effect remains at pH 12, indicating the complete unfolding of helical regions and their conversion into β-sheet-rich conformations.

References

1. DeFlores, L.P.; Ganim, Z.; Nicodemus, R.A.; Tokmakoff, A. Amide I′−II′ 2D IR Spectroscopy Provides Enhanced Protein Secondary Structural Sensitivity. *J. Am. Chem. Soc.* **2009**, *131*, 3385–3391, doi:10.1021/ja8094922.
2. Zandomeneghi, G.; Krebs, M.R.H.; McCammon, M.G.; Fändrich, M. FTIR Reveals Structural Differences between Native Β-sheet Proteins and Amyloid Fibrils. *Protein Science* **2004**, *13*, 3314–3321, doi:10.1110/ps.041024904.
3. Arnittali, M.; Tegopoulos, S.N.; Kyritsis, A.; Harmandaris, V.; Papagiannopoulos, A.; Rissanou, A.N. Exploring the Origins of Association of Poly(Acrylic Acid) Polyelectrolyte with Lysozyme in Aqueous Environment through Molecular Simulations and Experiments. *Polymers* **2024**, *16*, 2565, doi:10.3390/polym16182565.
